# A Review of the Reports on the Use Ehrlichs New Remedy for Syphilis

**Published:** 1910-10

**Authors:** Edgar G. Ballenger

**Affiliations:** Atlanta, Ga.


### A REVIEW OF THE REPORTS ON THE USE EHRLICHS NEW REMEDY FOR SYPHILIS.

By Edgar G. Ballenger, M. D., Atlanta, Ga.

Owing to the fact that our treatment for syphilis may have to be radically changed at an early date, if the remedy recently discovered by Ehrlich ultimately fulfills all that it now promises, I have decided to review the reports on this work to date rather than to present the paper I had planned to give when the title of my paper was placed on the program last January.

The tripod of scientific facts which have rendered Ehrlich's last discovery possible have been, first, the observation that syphilis was caused by the spirochaeta pallida; second, that apes and rabbits might be inoculated with syphilis; and finally, third, the Wassermann reaction, which, as a rule, may be depended upon to determine if syphilis has been cured.

Probably no scientific discovery has ever been presented with as many authoritative, favorable reports as has Ehrlich's 606. (This name was given to it because 605 preparations had been tested before the sucessful one was found; also, because the real name, dioxydiamidoarseno-benzol is such an unwieldly one.) While, of course, it is much too early to do more than guess at the finality of the alleged cures, one thing is certain, and that is that never before have we had a remedy which could be depended upon to act with such certainty and promptness in healing all syphilitic lesions and manifestations and in rendering the Wassermann reaction negative in such a short time—two to six weeks being within the usual limits. Many reports have shown this reaction remains negative, but the time is yet too short to say what will occur later.

That we have in 606 a specific for spirochetes seems certain; that we perhaps have in it the most remarkable remedy yet discovered seems not unreasonable to believe from the reports already made. There has been a remarkable unanimity of opinion in regard to the wonderful value of this preparation, and in the very small number of disagreeablbe bi-effects noted. Nothing seems more remarkable about 606 than its uniform adaptability and efficiency in all the stages and varieties of syphilis, even in the beginning of parasyphilitic affections.

Ehrlich claims the most important point about the remedy is that one injection usually destroys all of the germs and yet is comparatively harmless to animal tissues. What is more to the point is that this fact seems substantiated by nearly 2,500 clinical observations. Optic neuritis, which was sometimes caused by injections of atoxyl has not been caused by 606. Considerable pain is not infrequently produced at the site of the injection, and the temperature may rise at times to 104 degrees or 105 degrees. If given intravenously nausea, vomiting and fever may follow, but no pain is produced. It is urged that caution be exercised in giving this remedy, and this caution is of especial moment in patients with organic diseases, notably of the heart. Optic nerves and rational affections also contra-indicate its use. Neisse found chronic interstitial keratitis did not respond to this form of treatment—this seems to be the only syphilitic lesion not amenable to it.

Wechselmann has used this remedy in more than 600 patients and says erosive chancres become clean after twelve to twenty-four hours and rapidly heal; in pronounced induration the cleaning process is of the same rapidity, but absorption takes longer. Mucous patches of the mouth heal in from 24 to 48 hours, even if the patient is an inveterate smoker. The roseola disappears in a few days, as do malign ulcerous syphilides, rupia, gummata, and the small papulous syphilides which are otherwise so pertinaccious. Slower is the adisappearance of the large papulous syphilides, and for these a second injection becomes sometimes necessary. Osteocopic pains disappear as if by magic. Neisser says, after treating 126 patients, it is too early to draw final conclusions, as his earliest patients were dismissed as cured only about three months ago. He says further, that where visible symptoms of syphilis have existed they have disappeared in a manner which is absolutely astounding, and that he had thought it theoretically practically impossible that such inflammatory infiltrations would be so quickly absorbed and removed.

Meltzer says, "thousands of cases of syphilis have now been treated by many leading dermatologists of all shades of temperament, and all use one term to designate the effect; it is startling, stunning (verbluffend).

Jakimoff and Jakimona found the Ehrlich remedy, if the dose was sufficiently large, to be a powerful antidote in tick fever, which is caused by a spirochete somewhat like the spirochaeta pallida Iverson used this remedy in tertian malaria, and observed that a single injection caused a cessation of the symptoms and a disappearance of the plasmodia from the blood. He reports also that his results in the treatment were excellent. He thinks the most efficient method of administering it is a combination of the intramuscular and the intravenous routes. From 0.4 to 0.5 gramme is injected intravenously, and after 48 hours 0.3 gramme is injected intramuscularly. After the intravenous injections, within a few hours the temperature rises and there is a severe chill. He says the fever lasts but a few hours. Nausea and vomiting sometimes occur.

While Hoffman is skeptical of the ultimate cure, he reports some remarkable results in healing syphilitic affections. Case I, Congenital syphilis, with syphilitic ozena in a boy, fifteen years old. The symptoms presented were severe headache, unbearable foetor, exophthalmos, and paresis of the abducens. The symptoms disappeared almost immediatetly after the administration of 0.3 gramme of 606, and there was great subjective and objective improvement, but the Wassermann reaction was unchanged at the end of 16 days. Case II, Tertiary ulcerating syphilide of the face involving the nose, right cheek, upper and lower lips, strongly positive Wassermann reaction. Eighteen days after the injection there was perfect healing with the exception of an infiltrated border. He reports a case of pleurisy which he thinks was due to an embolic central pneumonia. Ehrlich believes the disagreeable bi-effects on the bladder and rectum noticed by Bohac and Sobotka were probably due to impurities in the methyl alcohol these observers used, as 132 other vials from this lot of 606 caused no such symptoms in the hands of others. Glueck notes particularly the rapidity of healing of syphilitic affections, and thinks the size of the dose has a special influence on the shortness of the healing process in the tertiary lesions. In a case of syphilitic arthritis complicated by periostitis of the tibia and gummata of the skin, not a trace of trouble was left at the end of six days. In two of his cases the results were not altogether satisfactory, and required a second dose.

Wechselmann describes his technic as follows: "Dioxydiamido-absenobenzol is dissolved by triturating in a mortar in from 1 to 2 cc of a soda solution, when, by adding acetic acid in drops, a fine yellow paste in precipitated. This precipitate is then made sterile and dissolved in, in from 1 to 2 cc of distilled water, and neutralized by the addition of 0.1 normal soda solution or a 1 per cent. acetic acid, according to the very carefully ascertained reaction with litmus paper. Absence of pain depends upon the exactness of this neutralization. The deposit is centrifugalized and the paste which has been deposited at the bottom of the glass is shaken up with a physiological sodium chloride solution. This mixture is drawn into a syringe and slowly injected subcutaneously below the shoulder blade in a place which has been made aseptic and treated with tincture of iodine."

Ehrlich's direction for the intravenous injection is as follows: "(a) Dioxydiamido-arsenobenzol 0.6 gramme, methyl alcohol 0.3 to 0.5 cc or 3 cc, glycol; (b) make another solution taking 240 or more cc of physiologic salt solution, 10.3 n 15 Na OH; amid continuous stirring, solutions 'a' and 'b' are mixed." This is then gradually injected into a vein.

The following are three interesting case histories from a recent report made by Wechselmann:

Case 1. A. P., twenty-three years of age, showed seven months ago the first effects of syphilis; four weeks later there appeared eczema. From November 28th, 1909, until the middle of March, 1910, he received thirty-five injections of mercury salicylate and calomel. For the past three months he had suffered from pains in both knee joints. On May 5, 1910, the patient was received into my wards in a very poor condition.

He was very much emaciated, and his skin had the appearance of that of a corpse. The face looked like a death's head, with an expression depicting the highest pain. The whole body and face were covered with ulcerations, incrusted, which reached through the skin into the subcutaneous tissue, together with scars. There was foetid smell from the nose. The septum was perforated; the left concha and vomer were ready to drop off. There were large ulcerations in the nasopharynx, and the uvula was destroyed in its left side. The patient could hardly swallow, on account of the extreme pain, and had to be fed by injections through a tube. The pulse was very small and of poor quality, 120 and over. We did not dare to give an injection, but, as his condition grew steadily worse, under iodipin injections, and as the end was to be expected in the very near future, 0.4 gramme of Ehrlich's 606 was injected on May 21st. The temperature did not rise and the pains did not amount to much. After two days there appeared a great improvement in his general condition, and on May 26th the beginning of a cure was everywhere apparent. On May 30tth the uvula had healed. The bony support of the nose had been thrown off in toto; the foetor had disappeared. On June 7th the patient could swallow and could walk around. His weight was on May 21st, 41.5 kilogrammes; on June 5th 42.5; and now 57 kilogrammes. He was discharged as cured.

Case 2. A very sensitive patient had suffered for five years from pains in the bones and used all kinds of treatment. He had a large swelling of the distal end of the ulna, which showed in the Rontgen picture a severe periostitis and an abscess filled with bony detritus. Although the intragluteal injection of Ehrlch's 606 was very painful, he stated that the night following it he had for the first time had a full night's rest.

Case 3. A second patient, who had severe nodular skin syphilides which could not be overcome with mercurial and arsacetin treatment, stated that for the last year and a half he had had such severe pains in the tibiae that he had injured his teeth in closing them while in pain. He stated that he had prevously suffered from a severe, very painful articular rheumatism, but that these pains could not be compared with the osteocopic pains. During the second night following the injection he was without pain.

It is undoubtedly too early to draw conclusions as to the permanency of the cures by dioxydiamido-arsenobensol, this time alone will prove. All patients should, of course, be closely watched for a number of years, and perhaps a repetition of the treatment occasionally during this time might be advisable.

I think permission to marry should be withheld at least until the patients have remained well for 2 or 3 years.

LITERATURE.

Fisher and Hoffe:—Munchener Med. Wochenschrift, July 5, 1910.

Hoffman:—Medizinische Klinic, Aug. 14, 1910.

Iverson:—Roussky Vratch, July 3d, 1910.

Loeb:—Munchener Med. Woch., July 26, 1910.

Meltzer:—New York Med. Jour., Aug. 20, 1910.

Michaelis:—Berliner Klin. Woch., July 25, 1910.

Nelson:—Laucet Clinic, Vol. CIV, No. 9.

Neisser:—Deutsche Med. Woch., June 30, 1910.

Scheiber & Hoppe:—Muchen Med. Woch., Juluy 5, 1910.

Treupel:—Deutsche Med. Woch., July 28 1910,

Wechselmann:—Berlin Klin Woch., July 4, 1910.

" Deutsche Med. Woch., July 28, 1910.

" Jour. Ama., Aug. 13, 1910.

" New York Med. Jour., Sept. 3, 1910.

